# Supplementary figures and images for: Comparison of two cable configurations in 3D printed steerable instruments for minimally invasive surgery
Source: PLoS One. 2022 Oct 4;17(10):e0275535. doi: 10.1371/journal.pone.0275535 (PMC9531805; doi:10.1371/journal.pone.0275535)

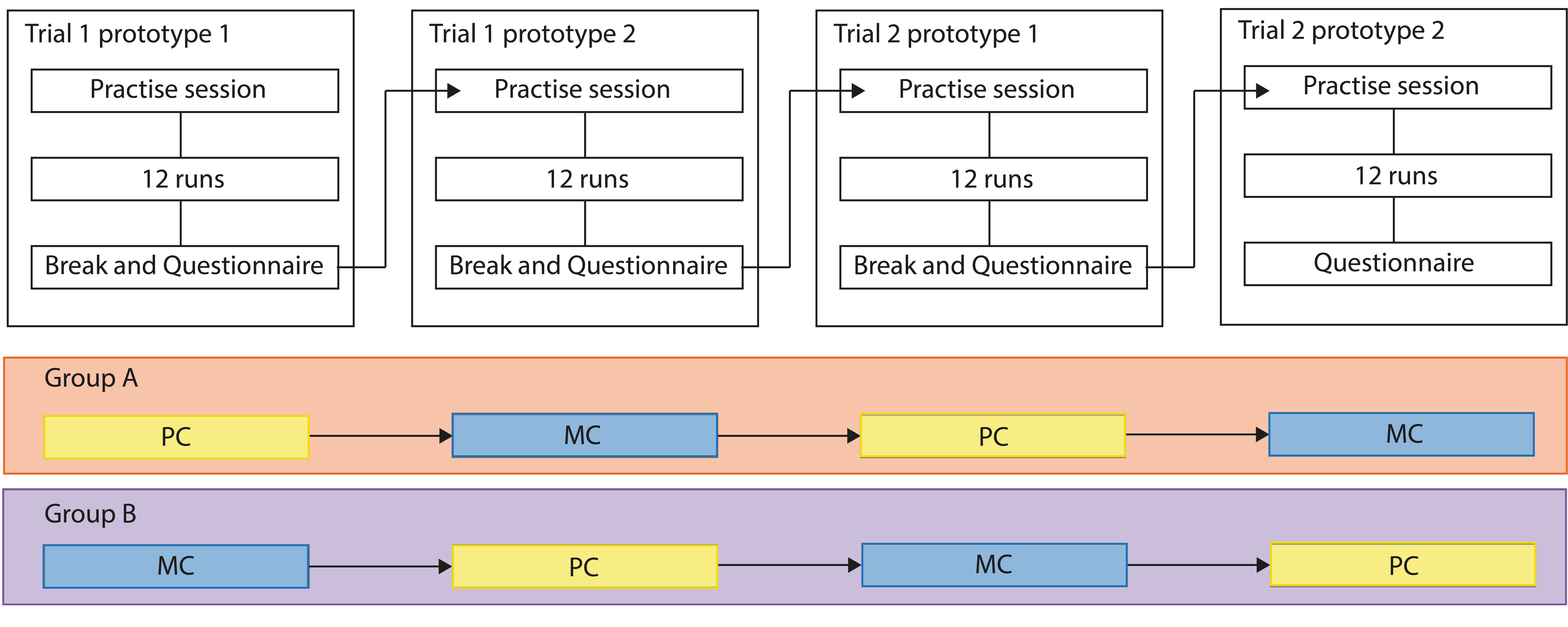

Supplement: S1 Fig — Each trial consists of 12 runs and the order of the instruments used for the two groups. Parallel configuration (PC), multi configuration (MC). (TIF) [file pone.0275535.s001.tif]
